# Priorities Wizard: Multisite Web-Based Primary Care Clinical Decision Support Improved Chronic Care Outcomes with High Use Rates and High Clinician Satisfaction Rates

**DOI:** 10.5334/egems.284

**Published:** 2019-04-03

**Authors:** JoAnn M. Sperl-Hillen, Rebecca C. Rossom, Elyse O. Kharbanda, Rachel Gold, Erik D. Geissal, Thomas E. Elliott, Jay R. Desai, D. Brad Rindal, Daniel M. Saman, Stephen C. Waring, Karen L. Margolis, Patrick J. O’Connor

**Affiliations:** 1 HealthPartners Institute, US; 2 Kaiser Permanente, US; 3 OCHIN, US; 4 Essentia Health, US

**Keywords:** clinical decision support, quality improvement, quality of care, population health, personalized medicine, patient centered care

## Abstract

**Introduction:**

Priorities Wizard is an electronic health record-linked, web-based clinical decision support (CDS) system designed and implemented at multiple Health Care Systems Research Network (HCSRN) sites to support high quality outpatient chronic disease and preventive care. The CDS system (a) identifies patients who could substantially benefit from evidence-based actions; (b) presents prioritized evidence-based treatment options to both patient and clinician at the point of care; and (c) facilitates efficient ordering of recommended medications, referrals or procedures.

**Methods:**

The CDS system extracts relevant data from electronic health records (EHRs), processes the data using Web-based clinical decision support algorithms, and displays the CDS output seamlessly on the EHR screen for use by the clinician and patient. Through a series of National Institutes of Health-funded projects led by HealthPartners Institute and the HealthPartners Center for Chronic Care Innovation and HCSRN partners, Priorities Wizard has been evaluated in cluster-randomized trials and expanded to include over 20 clinical domains.

**Results:**

Cluster-randomized trials show that this CDS system significantly improved glucose and blood pressure control in diabetes patients, reduced 10-year cardiovascular (CV) risk in high-CV risk adults without diabetes, improved management of smoking in dental patients, and improved high blood pressure identification and management in adolescents. CDS output was used at 71–77 percent of targeted visits, 85–98 percent of clinicians were satisfied with the CDS system, and 94 percent reported they would recommend it to colleagues.

**Conclusions:**

Recently developed EHR-linked, Web-based CDS systems have significantly improved chronic disease care outcomes and have high use rates and primary care clinician satisfaction.

## Introduction

Electronic health records (EHRs) have incontrovertibly improved documentation of medical care and facilitated care integration and evaluation of quality of care. Moreover, prompts and reminders delivered during office visits or other clinical encounters have improved rates of safety lab testing, laboratory monitoring of glycated hemoglobin (HbA1c) and lipids, and increased immunization rates and screening mammography rates [1]. However, there is much less evidence that EHR-linked clinical decision support (CDS) can improve important intermediate outcomes of chronic disease care, such as blood pressure (BP) control or glucose control in those with diabetes [2,3]. In some studies EHR-linked CDS has increased the frequency of testing lipids and glucose without improving lipid or glucose control, thus increasing the costs of care without clinical benefit.

This is a surprising state of affairs. In the early 1990s the Institute of Medicine and many experts predicted that introduction of outpatient EHRs would quickly improve chronic disease care, as well as other aspects of care [4,5]. However, challenges related to interoperability, data exchange, and EHR vendor reluctance to implement CDS beyond simple prompts and reminders have slowed broad dissemination of successful CDS systems to multiple care systems Moreover, because most EHR products do not take full advantage of data-consolidation and data-interpretation technology, the ability of EHRs to algorithmically gather, interpret and present useful clinical information during office visits is suboptimal. In a time-motion study, it took primary care clinicians four minutes and 52 mouse clicks to get 80 percent of the data needed to quantify a patient’s cardiovascular (CV) risk status and identify treatment options for uncontrolled CV risk factors [6].

## Methods

After analyzing failed outpatient CDS systems, our multisite (Table 1) and multidisciplinary research teams initiated a series of NIH-funded research projects (Table 2) beginning in 2006 to develop an effective clinician-designed EHR-linked Web-based CDS system that would be scalable to many care delivery systems. HealthPartners Medical Group in Minnesota first implemented this CDS system it in 2006, and randomized trial data that documented improved care were published in 2010 [7]. Since then the CDS system has been continuously updated and expanded. The current version of this CDS system, referred to as Priorities Wizard, addresses more than 20 clinical domains. (Table 3).

**Table 1:** Health Care Systems Research Network (HCSRN) Institutions Involved.

Institution	Role

HealthPartners Institute (HPI), Minneapolis, MN	Developed original CDS prototype in 2006, then expanded clinical domains using a series of funded NIH grants.
Kaiser Permanente Northwest, Portland, OR	Led a partnership with HPI and OCHIN to extend use of the CDS system to a large network of safety net clinics in 10 states.
Essentia Health, Duluth, MN	Partnered with HPI to include prediabetes, cancer prevention, and CV risk reduction in patients with serious mental illness.

**Table 2:** National Institute of Health Grants Supporting Clinical Decision Support (CDS) Development Across Multiple Clinical Domains (2006–2018).

NIH Institute	Grant	Principal Investigator	Clinical Domain

NIDDK	DK068314	O’Connor	Type 1 and Type 2 diabetes in adults
NHLBI	HL102144	O’Connor	Set of 6 major CV risk factors in adults
NHLBI	HL115082	Kharbanda	Elevated BP percentile and BMI percentile in teens
NHLBI	HL128614	Desai	Prediabetes management in adults
NIMH	MH092201	Rossom	CV risk in adults with serious mental illness
NCI	CA193396	Elliott	Screen for breast, colorectal, lung, and cervical cancer
NIDDK	DK118463	Sperl-Hillen	Identify and manage chronic kidney disease in adults
NHLBI	HL133793	Gold	Extend CV risk factor CDS to many safety net clinics
NHLBI	HL136937	Sperl-Hillen	Include adherence data to inform treatment options
NICHD	HD079463	Kharbanda	Assess appendicitis risk in tens with abdominal pain
NIDA	DA040316	Rossom/Bart	Opioid use disorder management in primary care

**Table 3:** Clinical Domains Currently Addressed in an Integrated Outpatient Clinical Decision Support System, and other clinical domains.

Current Clinical Domains	Future Clinical Domains

Type 2 Diabetes Type 1 Diabetes Prediabetes Chronic Kidney Disease Opioid Use Disorder Opioid Use in dental care Elevated BP in adolescents Elevated BMI in adolescents Hypertension Stage 1 Hypertension Stage 2 Dyslipidemias Tobacco/Nicotine Use Aspirin use 1° prevention Aspirin use 2° prevention Anticoagulation in atrial fibrillation Overweight in adults Obesity in adults Breast Cancer screening Colorectal Cancer screening Lung Cancer screening Cervical Cancer screening HPV Immunization Medication Adherence	Weight loss surgery Hypoglycemic risk Sleep apnea Asthma children* Asthma adults* COPD/Emphysema* Low Back Pain* Congestive Heart Failure* Depression* Anxiety* Physical Activity* Healthy Eating* Stress Management* Well-Being* Value-Based Care Learning Health Care System Social Determinants of Health*

* These depend upon being able to insert patient-reported data into CDS algorithms prior to the clinical encounter.

**Figure 1:**
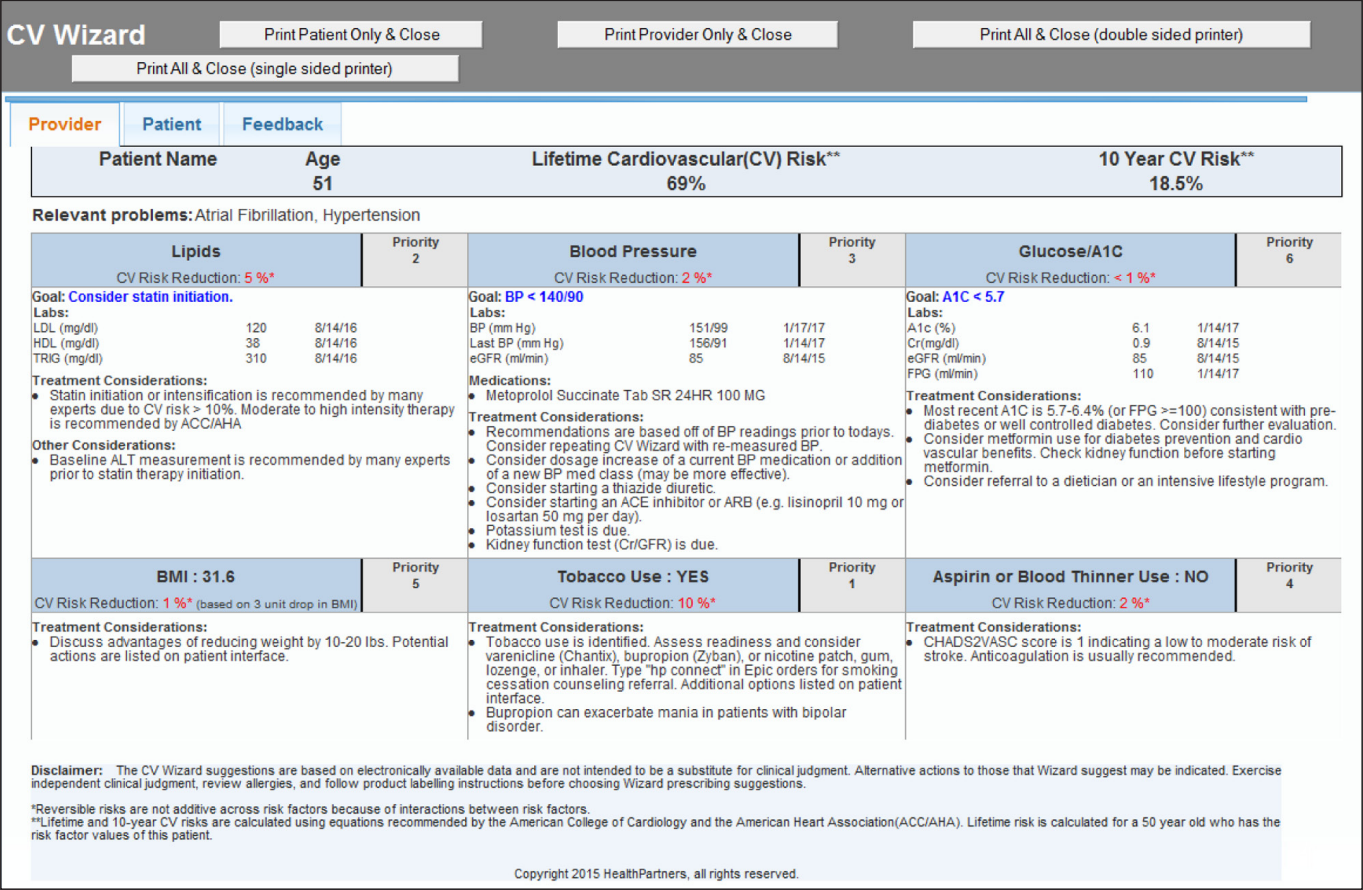
Screen shot of a clinician interface for a fictitious patient with prioritized treatment recommendations related to CV risk. This is printed by the rooming nurse and put on the exam room door for brief inspection by the clinician immediately before the visit, for visit planning purposes. It includes the 10-year atherosclerotic CV disease risk (risk of a heart attack or stroke in the next 10 years), amount of reversible CV risk, and personalized recommendations to consider for each domain. For example, smoking is the No. 1 priority for this patient; he could eliminate an absolute 10 percent of his 18.5 percent 10-year atherosclerotic CV disease risk if he quit. The CDS suggests consideration of counselling and medications to relieve cravings if he is ready to quit.

Key features of Priorities Wizard include the following:

- Identify patients who may benefit the most from diagnosis or changes in treatment. This is done by using current national guidelines for diabetes, hypertension, dyslipidemias, obesity, chronic kidney disease, atrial fibrillation, serious mental illness, prediabetes, tobacco use disorder, opioid use disorder, cancer prevention, immunizations and others.
- Identify evidence-based treatment options that may benefit a specific patient. The options are based on national evidence-based guideline recommendations and are specifically tailored to each patient based on demographics, current or previous treatments, distance from goal, allergies, comorbidities, and clinical considerations like renal, hepatic and cardiac function. Examples are shown in Figure 1.
- Prioritize evidence-based treatment options based on their potential benefit to the patient. As the patient’s clinical status evolves, new priorities will emerge. The CDS algorithms use risk prediction tools like the American College of Cardiology/American Heart Association (ACC/AHA) 10-year CV disease risk prediction equations, cancer risk assessment tools, and substance abuse screening and assessment methods. An example of prioritized CDS for CV risk factor control is shown in Figure 1.

## Results

### Clinical Outcomes

Published NIH-funded randomized trials show that this CDS system significantly improved glucose and blood pressure control in diabetes patients, reduced 10-year CV risk in high-CV risk adults without diabetes (Figure 3), improved smoking management in dental patients, and improved high blood pressure identification and management in adolescents [7,8,9,10,11].

### Impact on Primary Care Clinic Workflow

Priorities Wizard relies on clinic rooming staff, not clinicians, to trigger the CDS. Clinicians often do not complete suggested actions, but nurses and rooming staff usually do. Rooming staff can simply click the mouse twice to display and print the CDS.

**Figure 2:**
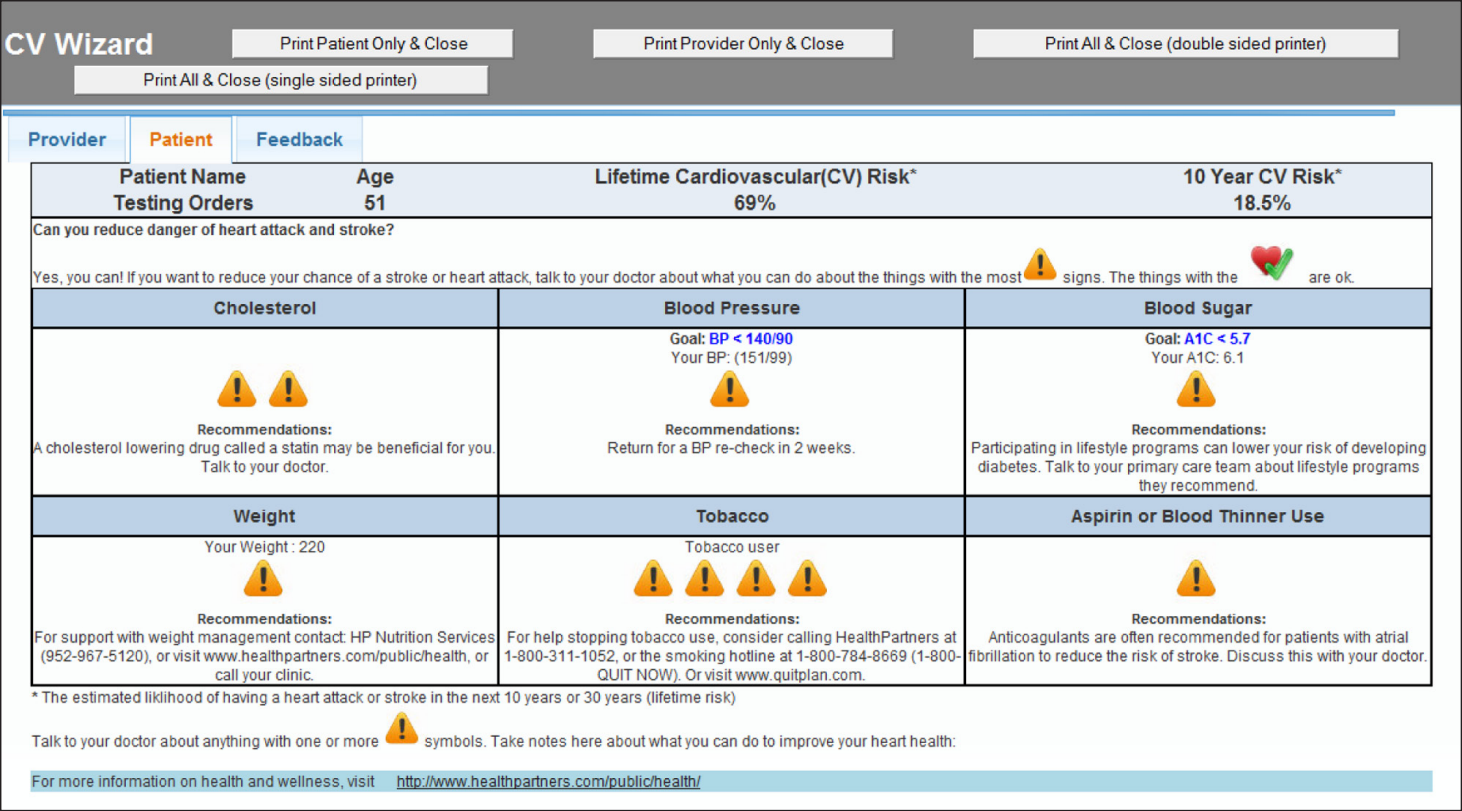
Screen shot of a patient interface for a different fictitious patient. This is printed by the rooming staff and handed to the patient before the start of the visit with the message, “The things with caution signs may reduce your danger of a stroke or heart attack. If you are interested in any of these things, you may want to talk to Dr. X about it today.” This patient has no CV risk factors at goal with tobacco being the highest priority followed by cholesterol treatment.

Priorities Wizard then presents prioritized information to the clinician and patient immediately before a clinical encounter. This supports patient-centered care, helps with visit planning, and engages the patient. Figure 2 shows an example of the printed CDS handout for a patient. Clinicians can also share the more quantitative clinician version with patients.

**Figure 3:**
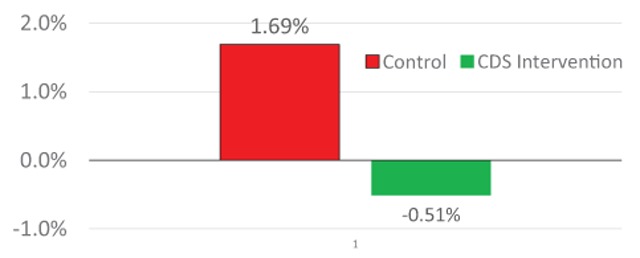
CV Wizard significantly reduced 10-year cardiovascular risk over the 14 month intervention period.

Priorities Wizard operates in real time with secure data transmission. Encrypted EHR data are sent securely to the Web service, where algorithms identify target patients, treatment options, and priorities. CDS output is then transmitted back to the EHR with a median Web service processing time of 300 milliseconds and display time of less than one second. Firewalls, double encryption, URL whitelisting, and Business Associate Agreements protect data and patient privacy (patient data cannot be used for purposes other than the care delivery specified in the agreement).

### CDS Use Rates

Primary care clinicians find the CDS useful, and they use it at 71–77 percent of targeted visits. Use rates are similar whether or not financial incentives are provided to clinicians or rooming staff to encourage high use rates, so we no longer deploy financial incentives. Training is done either by on-site face-to-face presentations by research staff over lunch, or by web-driven educational modules when these are the norm, as they often are at large rural medical systems that stretch across multiple states. It is gradually becoming apparent that face-to-face training over lunch for clinicians, rooming staff, and clinic managers is most effective, with supplemental web-based training for those unable to attend and for subsequently hired new staff. Training needs to be reinforced periodically. After training we provide automated feedback on CDS use rates by clinic name and clinician name to clinic managers and medical directors. In our experience this feedback, and clinic leadership endorsement of CDS use, are the two things most necessary to sustain high CDS use rates.

The CDS system we describe here is patient specific, not disease specific, to support high CDS use rates. Disease-specific CDS is less likely to be used because many patients have multiple comorbid conditions and primary care providers (PCPs) do not have time to trigger multiple disease-specific CDS tools at one visit. However, as more clinical domains are integrated into a single patient-centered CDS system, a higher proportion of visits will be targeted for CDS. As an example, targeting patients with high potential for CV risk reduction triggers CDS at about 20–25 percent of adult visits, but if those who need cancer screening are included, CDS will trigger at about 60 percent of adult visits. As the proportion of visits targeted for CDS increases, we have seen a downward trend in the percentage of targeted visits at which the CDS is actually viewed and used.

### Clinician Satisfaction with the CDS System

In surveys of clinicians who were randomized to use the CDS system, 94 percent said they would recommend it to colleagues, 98 percent believed it improved CV risk factor control in their patients, 93 percent reported it saved time when talking to patients about CV risk factor control, 95 percent stated it was helpful for shared decision making, 89 percent reported it influenced treatment recommendations, and 85 percent reported that their patients liked the CDS system. These high clinician satisfaction rates, based on clinician surveys with high response rates and presented in detail in a prior publication [9], have now been observed in multiple medical groups surveyed about this CDS system during randomized trials.

Compared to clinicians in clinics randomized to no CDS, those practicing in clinics with cardiovascular risk-related CDS reported significantly higher likelihood of discussing CV risk with patients and were more confident of the accuracy of the advice they provided.

### Business Case for CDS Use in Primary Care

This CDS system has been formally analyzed for its cost-effectiveness in adults with diabetes and is well within current guidelines for cost effectiveness with an estimated cost per quality-adjusted life year (QALY) gained of $3,017 in the base case scenario. This is a conservative estimate of cost-effectiveness in adults with diabetes because the costs were divided across only 20,000 diabetes patients, and the CDS system now guides the care of more than 100,000 diabetes patients, at essentially the same CDS operating costs [9]. However, these formal cost-effectiveness analyses done from the payer perspective do not capture the impact of CDS use on the primary care revenue cycle. CDS use does not increase frequency of clinic visits, but recent analyses (data not shown) suggest that primary care clinics randomized to use CDS had more complete diagnostic coding of the content of visits and higher levels of billing codes than clinics not using CDS. These preliminary data suggest that CDS use in primary care may have a favorable impact on primary care revenue generation [9].

In a project that targeted adults without diabetes or heart disease, but with high reversible CV risk, patients in the CDS intervention group had a statistically and clinically significant 2.3 percent absolute reduction in 10-year ACC/AHA CV risk. This would theoretically translate to 2,300 fewer heart attacks, strokes, or CV deaths per 100,000 eligible patients over 10 years, which, given the modest costs of CDS implementation and maintenance, would confer a return on investment (ROI) in the 2–4 range, depending on the cost of resources needed to achieve the better CV risk factor control, such as more statins and BP medications.

### Current Dissemination of the CDS system

Each day, this CDS system helps guide the care of more than 40,000 primary care patients age 5–75 years (9 million clinic visits a year) at 175 clinics in 15 care delivery systems located in 10 U.S. states The original Wizard CDS system addressed only glucose, BP, and lipid management in adults, but the current version developed with grant support from various NIH institutes (Table 2) has been extended in Priorities Wizard to provide CDS for over 20 clinical domains, including several in children or adolescents (Table 3).

One notable extension of the CDS system now guides PCPs in screening, diagnosis, and treatment of opioid use disorder (OUD). This OUD CDS module has been piloted by 55 clinicians at HealthPartners and Park Nicollet clinics in Minnesota, with plans to revise it and disseminate OUD CDS to three care delivery systems starting in 2019.

### Accelerating translation of evidence into practice

Research shows it can take many years to translate new evidence into practice [12]. However, Web-based CDS quickly deploys new evidence to the point of care. For example, CDS algorithms translated recent changes in blood pressure, lipid, and diabetes guidelines into routine practice within four months of guideline changes. Presenting evidence-based information to patients and clinicians using prioritized CDS makes it possible to influence care delivery quickly, making “informed shared decision making” a reality in routine primary care practice.

## Discussion

Newer EHR-linked, Web-based CDS systems have significantly improved chronic disease care and have high use rates and primary care clinician satisfaction. They accelerate the dissemination of new knowledge into practice, are cost effective and possibly cost saving at scale, and have already been disseminated to multiple large delivery systems. Privacy and data security have not been problematic, median web-site processing time is 300 milliseconds, and display time (from click to display) averages less than one second. The CDS screen includes a button enabling clinicians to provide immediate feedback to the informatics team if the CDS recommendations seem incomplete or implausible. While this feature has helped debug the algorithms over many years, in recent years it is often the clinician who needs debugging—often due to lack of familiarity with recent changes in evidence-based guidelines.

### Limitations

Despite these encouraging results and current rapid proliferation of EHR-linked web-based CDS systems to improve outpatient chronic disease care, the current technology has a number of important limitations. Although the positive impact on care outcomes is clinically and statistically significant, it is still a relatively modest clinical impact. This suggests that combining EHR-linked web-based CDS with other quality improvement interventions, such as case management or learning interventions may be the optimal way to address quality challenges [13].

Data privacy, confidentiality, and security are ongoing concerns. Even well-resourced health care systems with sophisticated and continuously vetted security measures can sometimes be hacked. In the face of ongoing security breaches in health care, banking and social media data bases, national laws to protect privacy and improve data security could inadvertently put major limits on the deployment of web-based CDS and other potentially beneficial health informatics technology.

Finally, there are financial costs to disseminating and maintaining CDS systems such as those described here. Experts hypothesize that the costs of CDS dissemination and maintenance will drop dramatically as interoperability improves, but inefficiencies in the current versions of FHIR and HL7 limit efficient dissemination of sophisticated real-time CDS systems. It currently takes four to eight months and a combined $60,000–$80,000 of programmer and clinician effort at donor and recipient sites to implement this CDS system in a new care delivery system. This is an obvious deterrent to widespread dissemination, and precludes implementation of such CDS systems at smaller clinics with limited programming resources, unless EHR vendors step up to the plate to help. EHR vendor reluctance to develop or to facilitate linkage to sophisticated web-based CDS systems represents a lost opportunity to improve quality of care.

### Future Challenges

Expand the CDS system to include additional clinical domains. Primary care clinicians deal with an average of more than four issues per visit, and an essential cornerstone of primary care CDS is to integrate multiple diseases or condition into a single CDS deployment, rather than offering multiple distinct disease-specific CDS tools that are used separately during a visit. Integration of CDS in a patient-centered way enables prioritization of recommendations based on potential benefits to a patient, and assures that recommendations are consistently patient-centered. Selected additional clinical domains suitable for inclusion in a primary care CDS system are listed in Table 3.

Develop more sophisticated methods to prioritize treatment options based on potential benefit to patient. As shown in Figures 1 and 2, it is straightforward to use CV risk prediction equations such as the ACC/AHA 10-year CVD risk equation which quantifies risk of a fatal or nonfatal heart attack or stroke in the next 10 years, to prioritize out-of-control CV risk factors. This can be done, for example, by running the ACC/AHA equation twice, once with the actual (elevated) SBP, and a second time to estimate the drop in CV risk based on what the anticipated effect on SBP would be after an additional BP-lowering medication were added, or a dose increased. This method of estimating benefits is imperfect because it (a) assumes that the clinical benefits accrue more rapidly than is actually the biological case, and (b) can only estimate a patient’s benefits based on prior experiences of large groups of patients. Imperfect as it may be, this method of estimating benefits is demonstrably better than the notoriously inaccurate and incomplete intuitive estimates of benefit and risk related to control of multiple CV risk factors made by most patients or clinicians.

Accurate prioritization of treatment options becomes even more challenging once other clinical domains such as opioid use disorder treatment, asthma management, or depression care are added to a CDS system. One formal way to prioritize treatment options based on their relative benefit to a patient would be to quantify benefits in terms of quality-adjusted life years. However, the impact on QALYs for many cancer prevention services is very small, especially in middle aged patients, and disclosure of the limited absolute risk reduction associated with many clinical services might have the unintended effect of lowering screening rates. On the other hand, patients have the right to know what the average benefits and risks of various clinical action are, even if benefits are small, and certainly when risks are high. Prioritization thus exposes many inherent ethical issues related to shared decision making. Evidence-informed decision making could lead many patients to decline beneficial treatment options or screening procedures based on large number needed to treat to achieve a benefit, or in the case of lung cancer screening due to the high rates of false positive results that trigger cascades of care that may confer substantive risks of serious side effects.

We have thus far focused more on potential benefit as a way to prioritize evidence-based clinical options. Another approach would be to prioritize based on a patient’s readiness to take action related to a particular clinical domain. Systematically eliciting a patient’s treatment preferences when multiple treatment options are available can be time consuming, but is important. Our approach to this is simply to show Figure 2 to the patient and say, “The actions with the caution signs may lower your danger of a stroke or heart attack. Are you interested in taking action on any of these things?” In surveys, clinicians who use this approach report that it saves time and patients like this approach. In this decision making framework, a patient who wants to continue smoking can immediately see estimates of the relative benefits of other actions that can be taken to reduce their CV risk.

An alternative method for prioritizing clinical domains would be to ask clinicians or patients to rank the relative importance of various clinical domains for certain general types of patients. Most primary care clinicians rank uncontrolled opioid use disorder, uncontrolled depression or serious mental illness, and current smoking as very high priorities. It is clear that much more work needs to be done in the area of prioritizing evidence-based care recommendations when multiple recommendations apply to a given patient.

Develop better ways to convey accurate information to patients of varying health literacy and numeracy levels. The clinician (Figure 1) and patient (Figure 2) interfaces have been part of successful CDS interventions. Both these formats were informed by extensive stakeholder input from primary care clinicians and patients, respectively. However, health literacy and numeracy vary greatly across patients (and statistical numeracy also varies across clinicians). Clinicians report that more mathematically or scientifically sophisticated patients (such as accountants, retired colonels, engineers, or nurses) prefer to take home the Figure 1 interface, which has more numbers. Also, some clinicians prefer to use the Figure 2 interface for both them and the patient, finding that the prioritization of clinical domains, rather than “what drug, what dose” advice is most valuable. It is clear that much more work needs to be done on how to most effectively communicate both risks and benefits to many types of patients, to support truly informed shared decision making. It is also clear that most clinicians and some patients find the printed interfaces to be a very useful visit planning tool.

Incorporate patient-reported information into the CDS algorithms. EHR interfaces can theoretically be used to input patient-reported data into the EHR, from which it could be sent to the CDS website for inclusion in CDS algorithms. Other ways to input patient-reported information include using text links, direct access to a CDS website, etc. Integration of pre-visit patient reported data with EHR data has revolutionary potential to extend primary care CDS to many additional clinical domains (Table 3). Lifestyle data could permit estimation of the health benefits of lifestyle changes on depression status, CV risk, weight management, or other dimensions of health. This area is rapidly evolving and some care delivery systems already have mechanisms in place to effectively collect and integrate patient-reported data. Incorporation of data from remote monitoring sources (such as home BP measures or home monitoring of blood glucose) is now available, but often not well-integrated with other data sources and therefore is difficult to incorporate into existing broad-based primary care CDS systems.

Learning Health Care System Applications. With proper data security measures (described above) and Business Associate Agreements, it may in the future be possible to store CDS output along with EHR data in off-line but frequently updated data warehouses where ongoing data analysis using machine learning, artificial intelligence (AI), and Bayesian or other analytic methods can produce new types of evidence to suggest optimal treatment strategies. Such an approach was recently reported by investigators in the HCSRN for assessing suicide risk, and it may soon be feasible to assess suicide risk using machine learning for every patient in the care delivery system [14].

Simpler innovations using archived data may be just as powerful. Archived CDS data has already demonstrated major clinician-level variation in hundreds of process of care measures that are identified for particular subsets of patients using the CDS algorithms. For example, there is threefold variation in primary care clinicians (10^th^ to 90^th^ percentile) in the proportion of their uncontrolled hypertension patients already on three or more hypertension meds, but not on a diuretic. Such data can precisely map quality and pinpoint messages that may be most relevant for every one of the 500 primary care clinicians in our medical group.

## Conclusion

Newer EHR-linked, Web-based CDS systems have significantly improved chronic disease care and have high use rates and primary care clinician satisfaction. Advances in data interoperability, data security, effective communication of risks and benefits to patients and clinicians, and integration of multiple clinical domains in a single patient-centered primary care CDS system will likely lead to further improvements in CDS systems with positive effects on both quality and cost of care in the near future.

## References

[1] Bright, TJ, Wong, A, Dhurjati, R, Bristow, E, Bastian, L, Coeytaux, RR, et al. Effect of clinical decision-support systems: A systematic review. Ann Intern Med. 2012; 157(1): 29–43. DOI: 10.7326/0003-4819-157-1-201207030-00450 22751758

[2] Roshanov, PS, Misra, S, Gerstein, HC, Garg, AX, Sebaldt, RJ, Mackay, JA, et al. Computerized clinical decision support systems for chronic disease management: A decision-maker-researcher partnership systematic review. Implementation science: IS. 2011; 6: 92 DOI: 10.1186/1748-5908-6-92 21824386 PMC3170626

[3] Roshanov, PS, Fernandes, N, Wilczynski, JM, Hemens, BJ, You, JJ, Handler, SM, et al. Features of effective computerised clinical decision support systems: Meta-regression of 162 randomised trials. BMJ. 2013; 346: f657 DOI: 10.1136/bmj.f657 23412440

[4] Institute of Medicine. The computer-based patient record. An essential technology for health care. 1991; 190 Washington DC: National Academy Press.

[5] Institute of Medicine. Guidelines for clinical practice. From development to use. 1992; 426 Washington DC: National Academy Press. 25121254

[6] Koopman, RJ, Kochendorfer, KM, Moore, JL, Mehr, DR, Wakefield, DS, Yadamsuren, B, et al. A diabetes dashboard and physician efficiency and accuracy in accessing data needed for high-quality diabetes care. Annals of family medicine. 2011; 9(5): 398–405. DOI: 10.1370/afm.1286 21911758 PMC3185474

[7] O’Connor, PJ, Sperl-Hillen, JM, Rush, WA, Johnson, PE, Amundson, GH, Asche, SE, et al. Impact of electronic health record clinical decision support on diabetes care: A randomized trial. Annals of family medicine. 2011; 9(1): 12–21. DOI: 10.1370/afm.1196 21242556 PMC3022040

[8] Gilmer, TP, O’Connor, PJ, Sperl-Hillen, JM, Rush, WA, Johnson, PE, Amundson, GH, et al. Cost-effectiveness of an electronic medical record based clinical decision support system. Health services research. 2012; 47(6): 2137–58. DOI: 10.1111/j.1475-6773.2012.01427.x 22578085 PMC3459233

[9] Sperl-Hillen, JM, Crain, AL, Margolis, KL, Ekstrom, HL, Appana, D, Amundson, GH, et al. Clinical decision support directed to primary care patients and providers reduces cardiovascular risk: A randomized trial. J Am Med Inform Assoc. 2018; 25(9): 1137–46. DOI: 10.1093/jamia/ocy085 29982627 PMC6658854

[10] Rindal, DB, Rush, WA, Schleyer, TK, Kirshner, M, Boyle, RG, Thoele, MJ, et al. Computer-assisted guidance for dental office tobacco-cessation counseling: A randomized controlled trial. American journal of preventive medicine. 2013; 44(3): 260–4. DOI: 10.1016/j.amepre.2012.10.023 23415123 PMC3579569

[11] Kharbanda, EO, Asche, SE, Sinaiko, AR, Ekstrom, HL, Nordin, JD, Sherwood, NE, et al. Clinical Decision Support for Recognition and Management of Hypertension: A Randomized Trial. Pediatrics. 2018 2; 141(2). DOI: 10.1542/peds.2017-2954 PMC5810603 29371241

[12] Rogers, EM. Diffusion of Innovations 5th ed. New York: Free Press; 2003.

[13] Renders, CM, Valk, GD, Griffin, SJ, Wagner, EH, Van Eijk, JT and Assendelft, WJ. Interventions to improve the management of diabetes in primary care, outpatient, and community settings: A systematic review. Diabetes care. 2001; 24(10): 1821–33. DOI: 10.2337/diacare.24.10.1821 11574449

[14] Simon, GE, Johnson, E, Lawrence, JM, Rossom, RC, Ahmedani, B, Lynch, FL, et al. Predicting Suicide Attempts and Suicide Deaths Following Outpatient Visits Using Electronic Health Records. Am J Psychiatry; 2018 DOI: 10.1176/appi.ajp.2018.17101167 PMC6167136 29792051

